# Wilms’ tumor gene 1 is an independent prognostic factor for pediatric acute myeloid leukemia following allogeneic hematopoietic stem cell transplantation

**DOI:** 10.1186/s12885-021-08022-0

**Published:** 2021-03-19

**Authors:** Dao-Xing Deng, Juan-Juan Wen, Yi-Fei Cheng, Xiao-Hui Zhang, Lan-Ping Xu, Yu Wang, Chen-Hua Yan, Yu-Hong Chen, Huan Chen, Wei Han, Feng-Rong Wang, Jing-Zhi Wang, Ya-Zhen Qin, Kai-Yan Liu, Xiao-Jun Huang, Xiao-Su Zhao, Xiao-Dong Mo

**Affiliations:** 1 Peking University People’s Hospital, Peking University Institute of Hematology, National Clinical Research Center for Hematologic Disease, Research Unit of Key Technique for Diagnosis and Treatments of Hematologic Malignancies, Chinese Academy of Medical Sciences, 2019RU029, Beijing Key Laboratory of Hematopoietic Stem Cell Transplantation, Beijing, China; 2 Department of Hematology, Peking University Shenzhen Hospital, Shenzhen, China; 3 Peking-Tsinghua Center for Life Sciences, Beijing, China; 4 Collaborative Innovation Center of Hematology, Peking University, Beijing, China

**Keywords:** Pediatric, Acute myeloid leukemia, Allogeneic hematopoietic stem cell transplantation, Wilms’ tumor gene 1, Relapse

## Abstract

**Background:**

Sequential monitoring of Wilms’ tumor gene 1 (WT1) after allogeneic hematopoietic stem cell transplantation (allo-HSCT) could predict relapse in adult acute myeloid leukemia (AML). However, the prognostic role of WT1 in pediatric AML after allo-HSCT is unclear. Thus, we determined to see whether sequential monitoring of WT1 after allo-HSCT could predict relapse in AML children.

**Methods:**

Pediatric AML patients receiving allo-HSCT from January 21, 2012 to December 20, 2018 at the Peking University Institute of Hematology were included in this study. WT1 expression level was determined by TaqMan-based reverse transcription-polymerase chain reaction. WT1 sequential monitoring was performed 1, 2, 3, 4.5, 6, 9, and 12 months post-transplantation and at 6-month intervals thereafter. The primary end point was relapse. The secondary end points included disease-free survival (DFS), overall survival (OS), and non-relapse mortality (NRM). Kaplan–Meier analysis was used for DFS and OS estimates, while competing risk analysis was used for estimating relapse and NRM.

**Results:**

Of the 151 consecutive patients included, the median age was 10 years (range, 1–17). The optimal cutoff value of WT1 within 1 year after allo-HSCT to predict relapse was 0.8% (80 WT1 copies/10^4^ ABL copies), with a sensitivity of 60% and specificity of 79%. Compared with WT1 expression < 0.8%, WT1 expression ≥0.8% indicated significantly higher 5-year cumulative incidence of relapse (CIR, 35.1% vs. 11.3%; *P* = 0.001), lower 5-year disease-free survival (DFS, 60.4% vs. 80.8%; *P* = 0.009), and lower 5-year overall survival (OS, 64.9% vs. 81.6%; *P* = 0.038) rates. Multivariate analyses showed that WT1 was an independent risk factor for relapse (HR 2.89; 95% confidence interval (CI), 1.25–6.71; *P* = 0.014). Both the CIR (5-year CIR: 8.3% vs. 11.3%; *P* = 0.513) and DFS (5-year DFS: 91.7% vs. 80.8%; *P* = 0.208) were comparable between patients achieving minimal residual disease (MRD) negativity after preemptive interferon-α (IFN-α) treatment and those without MRD after allo-HSCT, which were better than those of MRD-positive patients without preemptive therapies.

**Conclusions:**

Sequential monitoring of WT1 could predict relapse in pediatric AML after allo-HSCT. WT1-directed immunotherapy may have the potential to prevent relapse and improve survival.

**Supplementary Information:**

The online version contains supplementary material available at 10.1186/s12885-021-08022-0.

## Background

Allogeneic hematopoietic stem cell transplantation (allo-HSCT) is one of the most critical therapies for pediatric acute myeloid leukemia (AML) patients, and many of them can achieve long-term disease-free survival (DFS) [1, 2]. However, relapse remains the most important cause of transplantation failure [3]. Therefore, preventing relapse is important to improve the outcomes of pediatric AML patients receiving allo-HSCT.

Minimal residual disease (MRD) monitoring is the most important method to recognize the early presentations of relapse, which could help to prevent post-transplant relapse [4]. MRD monitoring methods include multiparameter flow cytometry (MFC) and polymerase chain reaction (PCR) [5]. MFC monitoring is critical for AML patients, but AML carries multiple immunophenotypic clones and frequently changes the antigen profile after therapies, which restricts the application of MFC-MRD in AML patients [6]. PCR is another important method for MRD monitoring, particularly for those with leukemic fusion genes [7]. Currently, *PML-RARα*, *RUNX1-RUNX1T1*, *CBFβ–MYH11*, and *NPM1* mutation are recommended for use as molecular markers for MRD monitoring [5]; however, only 30–40% of pediatric AML patients have these molecular markers [8]. Therefore, for patients without mature molecular MRD markers, ways to monitor MRD should be further identified.

Wilms’ tumor gene 1 (WT1) is highly expressed in most acute leukemia patients and can be quantitatively detected, enabling it to be a potential MRD marker for pediatric AML without special molecular markers [7]. Some studies have shown that WT1 could predict relapse in AML children receiving chemotherapy [9, 10]. In addition, some studies showed that WT1 could predict post-transplant relapse of AML [11–14], but the samples of children enrolled in these studies were small. Meanwhile, some authors observed that the expression of WT1 was different between children and adults [15]. In a word, for relapse prediction of pediatric AML after allo-HSCT, data of WT1 expression was in urgent need. To the best of our knowledge, only one study explored the role of WT1 expression in relapse prediction in pediatric AML after allo-HSCT [16]. This study found that WT1 failed to predict relapse in pediatric AML after allo-HSCT. However, this study only involved 2 time points (+42d and + 100d) of WT1 monitoring. In addition, previous studies showed that sequential monitoring of WT1 after allo-HSCT could predict relapse in adult AML patients [11, 12]. Therefore, because of insufficient time points, the conclusion of meaningless WT1 monitoring in pediatric AML after allo-HSCT could not be made. To sum up, WT1 sequential monitoring was critical to predict relapse in AML patients after allo-HSCT, but whether sequential monitoring of WT1 could predict relapse of AML children after allo-HSCT was unclear.

Therefore, we aimed to identify the prognostic value of WT1 sequential monitoring in pediatric AML patients after allo-HSCT. In particular, we wanted to identify the cut-off value of WT1 for predicting relapse after allo-HSCT in AML children.

## Methods

### Patients

From January 21, 2012 to December 20, 2018, 151 consecutive pediatric AML patients receiving allo-HSCT at the Peking University Institute of Hematology (PUIH) were enrolled in this study with the following criteria: (1) < 18 years old; (2) diagnosed with AML without *PML-RARα, RUNX1-RUNX1T1, CBFβ–MYH11,* or *NPM1* mutation; and (3) monitored WT1 expression regularly after allo-HSCT. This study followed the principles of the Helsinki Declaration and was approved by the ethical committee of Peking University People’s Hospital.

### Transplantation regimens

The preconditioning regimens consisted of cytarabine (Ara-C), busulfan, cyclophosphamide, and simustine. Rabbit anti-thymocyte globulin was administered to the HLA-unrelated donor (URD), HLA-haploidentical related donor (haplo-RD), and umbilical cord blood (UCB) donor groups (eMethods in the Supplement) [17–19]. Patients received cyclosporine A (CsA), mycophenolate mofetil (MMF), and short-term methotrexate (MTX) as GVHD prophylaxis. UCB transplantation recipients received methylprednisolone (MP) instead of MTX [19, 20]. Donor selection, HLA typing, and stem cell harvesting have been described in detail elsewhere [21].

### MRD monitoring and definition

MRD monitoring was based on leukemia-associated aberrant immune phenotypes (LAIPs) detected by MFC [22] and WT1 expression levels determined through TaqMan-based RQ-PCR technology (eMethods in the Supplement) [11]. MFC positivity was defined as > 0.1% of cells with a LAIP in post-transplantation bone marrow (BM) samples according to the ELN criteria [5]. The transcript level was calculated as WT1 transcript copies/ABL copies in percentage. According to our experiment method, the transcript level of WT1 could also be calculated as WT1 copies/10^4^ ABL copies. Routine MRD monitoring was performed 1, 2, 3, 4.5, 6, 9, and 12 months post-transplantation and at 6-month intervals thereafter. MRD positivity was defined as MFC positivity or WT1 positivity. WT1 positivity alone (WT1+ alone) was defined as WT1 positivity without MFC positivity in BM samples. Combined MRD positivity (MRDco+) was defined as both MFC positivity and WT1 positivity in a BM sample. Relapse was defined as the recurrence of > 5% BM blasts, the reappearance of blasts in the blood or the development of extramedullary disease. We defined WT1 positivity except relapse. The data of WT1 expression at the time of relapse would not be considered.

### Preemptive intervention

A part of patients included in the study ever entered a prospective clinical study exploring the efficacy of interferon-α (IFN-α) treatment from 2012 to 2014, and results of the study had been published [23]. Since then, IFN-α treatment was a routine for patients with MRD positivity at our center. Moreover, IFN-α treatment was also one of the preemptive interventions recommended by the consensus about treatment and prevention of leukemia relapse after allo-HSCT in China [3]. Chemotherapy plus donor lymphocyte infusion (Chemo-DLI) was a routine for patients with MRD positivity at our center since 2011 [24]. The protocol of IFN-α treatment [23, 25–28] and Chemo-DLI [24, 29, 30] was summarized in the eMethods in the Supplement. Informed consent to preemptive intervention was gotten from all patients’ guardian. In addition, 26 patients with WT1 positivity did not receive preemptive intervention. Among them, two patients had active aGVHD, five patients had active cGVHD, two patients had active infections, and 17 patients refused to receive preemptive intervention since the prognostic role of WT1 expression was uncertain in pediatric AML after allo-HSCT before this study.

### Statistical methods

The last follow-up date was 31 December 2019. The primary end point was relapse. The secondary end points included DFS, overall survival (OS), and non-relapse mortality (NRM). Relapse, DFS, OS and NRM were calculated from the date of transplantation. Relapse was defined as the recurrence of > 5% BM blasts, the reappearance of blasts in the blood or the development of extramedullary disease. Deaths were the events for OS. The events for DFS included relapse and death of any cause. Kaplan–Meier analysis was used for DFS and OS estimates, with the log-rank test used for comparisons between groups. Competing risk analysis was used for estimating relapse and NRM, and the Gray’s test was applied for comparisons between subgroups. NRM was the competing event for relapse and vice versa. Cox’s proportional hazards model was used for multivariable analyses. *P* < 0.05 was considered statistically significant. Analyses were performed using SPSS 20.0 (Mathsoft, Seattle, WA), GraphPad Prism 6 (GraphPad Software Inc., La Jolla, CA) and R software (http://cran.R-project.org).

## Results

### Patient characteristics

A total of 151 consecutive pediatric AML patients were enrolled (Table 1). The median age was 10 years (range, 1–17) and the median follow-up time was 798 days (range, 55–2901) after allo-HSCT. Thirty-one patients experienced relapse, and nine patients suffered TRM. In total, we collected 1242 BM samples from these patients after allo-HSCT.

**Table 1:** Patient characteristics (*n* = 151)

Characteristics	***N*** = 151
Gender (male/female)	89/62
Median age (years, range)	10 (1–17)
FAB type	
M0	5
M1	4
M2	43
M4	17
M5	57
M6	11
M7	10
MDS-AML	3
Therapy-related AML	1
Cytogenetics and molecular abnormalities	
Intermediate	94
Unfavorable	57
Disease status before allo-HSCT	
CR1	132
CR2	15
NR	4
Donor type	
ISD	13
URD	5
Haplo-RD	129
UCB	4
Median MNC (×10^8/kg, range)	8.57 (0.59–16.66)
Median CD34 (×10^6/kg, range)	2.85 (0.17–10.95)
NE engraftment (n, %)	151 (100.0%)
Median time from HSCT to NE engraftment (days, range)	12 (10–23)
PLT engraftment (n, %)	146 (96.7%)
Median time from HSCT to PLT engraftment (days, range)	15.5 (7–128)

*CR* Complete remission, *NR* No remission, *ISD* HLA-identical sibling donor, *URD* HLA-unrelated donor, *Haplo-RD* HLA-haploidentical related donor, *UCB* Umbilical cord blood, *MNC* Mononuclear cell, *NE* Neutrophil, *PLT* Platelet

### The expression of WT1 in patients without relapse after allo-HSCT

**Fig. 1:**
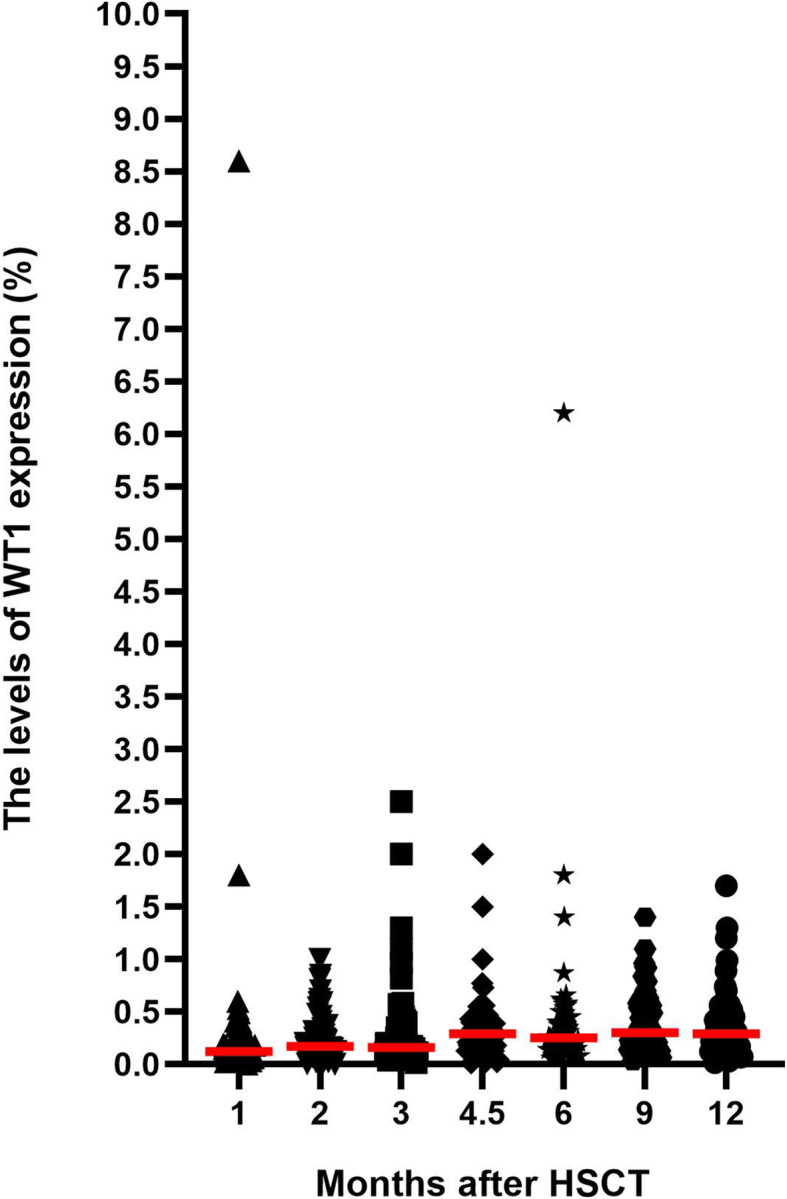
WT1 expression at different points after allo-HSCT in patients maintaining CR without interventions (*n* = 81). Horizontal bars show the median values of WT1 at each time point

A total of 81 patients did not receive any intervention and showed persistent complete remission (CR), and 501 BM samples were collected from these patients within 1 year after allo-HSCT. The median WT1 expression levels at + 1, + 2, + 3, + 4.5, + 6, + 9, and 12 months were 0.120% (0.000–8.600%), 0.170% (0.000–1.000%), 0.160% (0.016–2.500%), 0.290% (0.014–2.000%), 0.250% (0.059–6.200%), 0.300% (0.035–1.400%), and 0.290% (0.016–1.700%), respectively (Fig. 1).

### The cutoff value of WT1 for relapse prediction

The highest expression level of WT1 within 1 year after allo-HSCT was the highest one in seven time points (1, 2, 3, 4.5, 6, 9, and 12 months post-transplantation). If a patient relapsed within 1 year after allo-HSCT, only the values of WT1 expression before relapse would be used for picking out the highest expression level of WT1. We carried out ROC analysis to explore the association between relapse and the highest expression level of WT1 within 1 year after allo-HSCT in 96 patients who did not receive any preemptive interventions. The area under the ROC curve was 0.697 (95% confidence interval (CI) =0.541–0.854, *P* = 0.016; eFigure 1 in the Supplement). The optimal cutoff value of WT1 within 1 year after HSCT to predict relapse was 0.8% (80 WT1 copies/10^4^ ABL copies), with a sensitivity of 60% and specificity of 79% (eTable 1 in the Supplement).

### The association between WT1 and MFC positivity within 1 year after allo-HSCT

We defined WT1 positivity as WT1 ≥ 0.8%. Spearman correlation analysis showed that WT1 positivity was significantly associated with MFC positivity (Spearman’s correlation coefficient: 0.344, *P* < 0.001) within 1 year after allo-HSCT. Among the 67 patients with WT1 positivity, 9 showed WT1 and MFC positivity simultaneously. Four patients showed WT1 positivity 28, 42, 148, and 244 (median: 95) days prior to MFC positivity, respectively.

### WT1 positivity within 1 year after allo-HSCT predicted poor outcomes

**Fig. 2:**
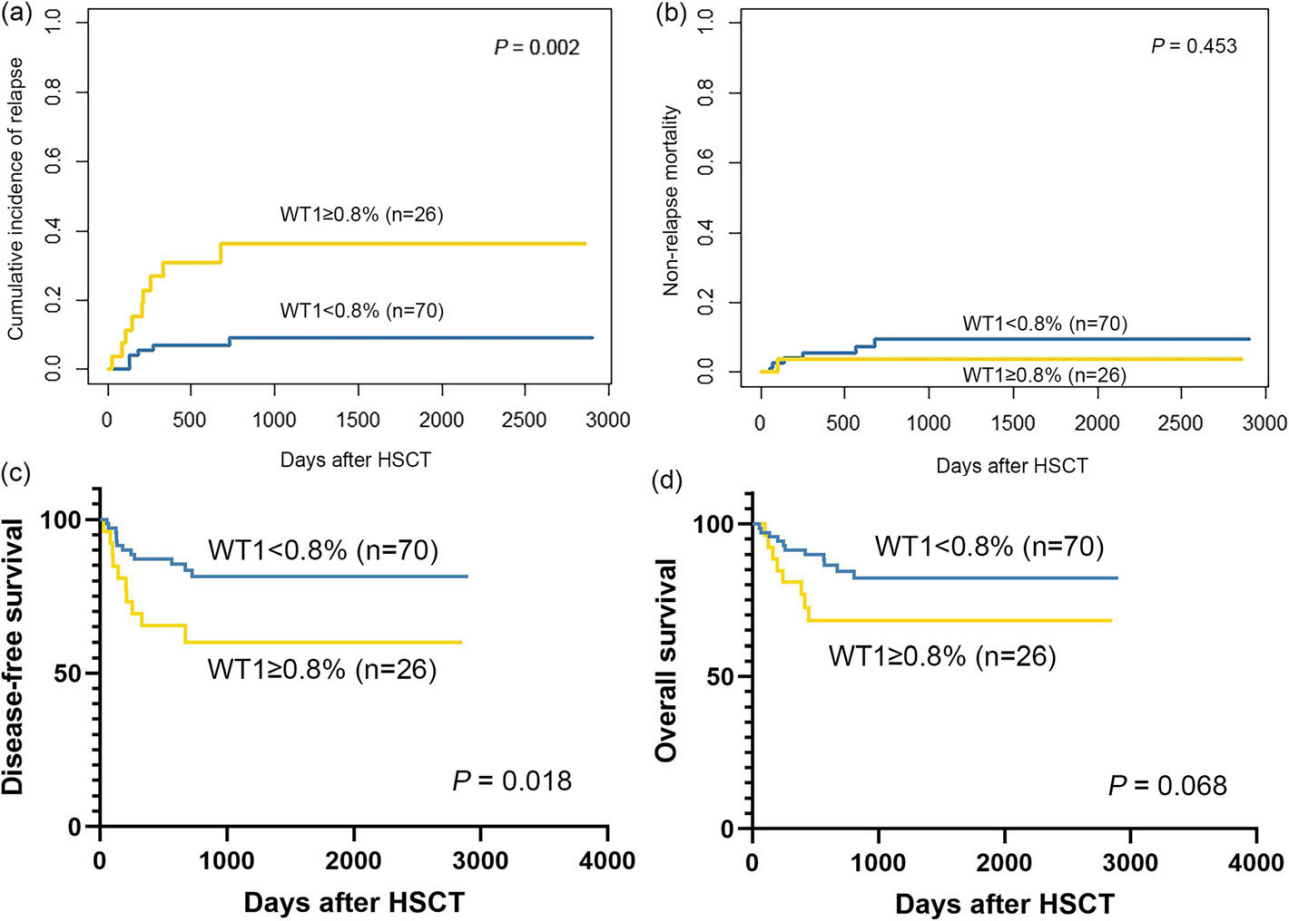
The outcomes of patients without preemptive interventions according to WT1 after allo-HSCT (*n* = 96): **a** relapse, **b** non-relapse mortality, **c** disease-free survival, and **d** overall survival

**Fig. 3:**
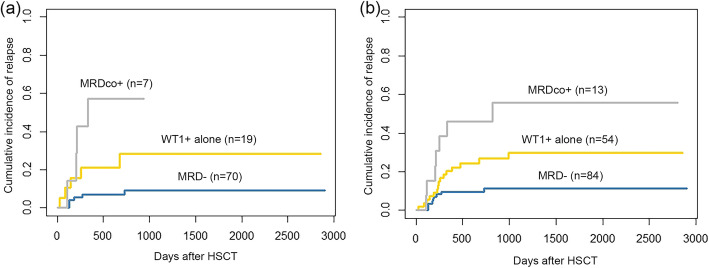
The relapse of patients according to MRD status after allo-HSCT. **a** 2-year CIR in patients without any preemptive interventions (*n* = 96): WT1+ alone vs. MRD-: 28.4% vs. 9.2%, *P* = 0.032; MRDco+ vs. MRD-: 57.1% vs. 9.2%, *P* < 0.001; WT1+ alone vs. MRDco+: 28.4% vs. 57.1%; *P* = 0.168. **b** 5-year CIR in all patients (*n* = 151): WT1+ alone vs. MRD-: 29.8% vs. 11.3%, *P* = 0.013; MRDco+ vs. MRD-: 55.8% vs. 11.3%, *P* < 0.001; WT1+ alone vs. MRDco+: 29.8% vs. 55.8%, *P* = 0.060

Among the patients without preemptive interventions after allo-HSCT (*n* = 96), patients with WT1 positivity within 1 year after allo-HSCT had a significantly higher 2-year cumulative incidence of relapse (CIR, 36.2% vs. 9.2%; *P* = 0.002), a significantly lower 2-year probability of DFS (59.9% vs. 81.4%; *P* = 0.018), and a trend of a lower 2-year probability of OS (68.2% vs. 84.5%; *P* = 0.068) than those with WT1 negativity (Fig. 2). The 2-year CIR of patients with WT1+ alone (28.4% vs. 9.2%; *P* = 0.032) and MRDco+ (57.1% vs. 9.2%; *P* < 0.001) were both significantly higher than that of those without MRD. In addition, the 2-year CIR was comparable between the WT1+ alone and MRDco+ groups (28.4% vs. 57.1%; *P* = 0.168) (Fig. 3a). Multivariate analyses showed that WT1 positivity after allo-HSCT was an independent risk factor for relapse (HR 6.15; 95% confidence interval (CI), 1.62–23.33; *P* = 0.008) and DFS (HR 2.97; 95% CI, 1.05–8.46; *P* = 0.041) (Table 2).

**Table 2:** Multivariate analyses for 2-year outcomes in patients without preemptive interventions (*n* = 96)

Variables	HR (95% CI)	***P*** value
Relapse		
Cytogenetics and molecular abnormalities		
Intermediate	1.00	
Unfavorable	3.41 (1.08–10.81)	0.037^*^
Disease status before allo-HSCT		
CR1	1.00	
CR2	2.03 (0.39–10.43)	0.398
NR	114.16 (13.86–940.41)	< 0.001^*^
MRD status after allo-HSCT		
MRD-	1.00	
WT1+ alone	6.15 (1.62–23.33)	0.008^*^
MRDco+	14.70 (3.63–59.58)	< 0.001^*^
DFS		
Cytogenetics and molecular abnormalities		
Intermediate	1.00	
Unfavorable	2.88 (1.15–7.20)	0.024^*^
Disease status before allo-HSCT		
CR1	1.00	
CR2	1.11 (0.24–5.04)	0.896
NR	33.06 (5.94–184.00)	< 0.001^*^
MRD status after allo-HSCT		
MRD-	1.00	
WT1+ alone	2.97 (1.05–8.46)	0.041^*^
MRDco+	6.37 (1.94–20.99)	0.002^*^
OS		
Cytogenetics and molecular abnormalities		
Intermediate	1.00	
Unfavorable	2.90 (1.10–7.66)	0.031^*^
Disease status before allo-HSCT		
CR1	1.00	
CR2	0.47 (0.06–3.66)	0.472
NR	14.97 (2.95–76.02)	0.001^*^
MRD status after allo-HSCT		
MRD-	1.00	
WT1+ alone	1.62 (0.49–5.42)	0.433
MRDco+	7.13 (2.11–24.07)	0.002^*^

*CR* Complete remission, *NR* No remission, *MRD-* MRD negativity, *WT1+ alone* WT1 positivity alone, *MRDco+* Combined MRD positivity, *DFS* Disease-free survival, *OS* Overall survival, *HR* Hazard ratio, *CI* Confidence interval

* means *P* < 0.05

In total, 22 patients with WT1 positivity within 1 year after allo-HSCT suffered relapse. The median interval from WT1 positivity to relapse was 105 (13–714) days. In addition, seven patients with MFC and WT1 positivity (MRDco+) after allo-HSCT suffered relapse. The median interval from MRDco+ to relapse was 58 (13–550) days. In the total cohort (*n* = 151), patients with WT1 positivity within 1 year after allo-HSCT had a significantly higher 5-year CIR (35.1% vs. 11.3%; *P* = 0.001), a lower 5-year DFS (60.4% vs. 80.8%; *P* = 0.009), and a lower 5-year OS (64.9% vs. 81.6%; *P* = 0.038) than those with WT1 negativity (eFigure 2 in the Supplement). The 5-year CIR of patients with WT1+ alone (29.8% vs. 11.3%; *P* = 0.013) and MRDco+ (55.8% vs. 11.3%; *P* < 0.001) were both significantly higher than that of those without MRD. In addition, the 5-year CIR of patients with WT1+ alone tended to be lower than that of patients with MRDco+ (29.8% vs. 55.8%; *P* = 0.060) (Fig. 3b). Multivariate analyses showed that WT1 positivity after allo-HSCT was an independent risk factor for relapse (HR 2.89; 95% CI, 1.25–6.71; *P* = 0.014) (Table 3).

**Table 3:** Multivariate analyses for 5-year outcomes in the total patients (*n* = 151)

Variables	HR (95% CI)	***P*** value
Relapse		
Cytogenetics and molecular abnormalities		
Intermediate	1.00	
Unfavorable	2.23 (1.05–4.75)	0.037^*^
Disease status before allo-HSCT		
CR1	1.00	
CR2	1.93 (0.55–6.81)	0.306
NR	10.74 (3.25–35.47)	< 0.001^*^
Donor type		
ISD	1.00	
URD	0.32 (0.04–2.82)	0.302
Haplo-RD	0.33 (0.12–0.90)	0.030^*^
UCB	1.85 (0.35–9.86)	0.472
MRD status after allo-HSCT		
MRD-	1.00	
WT1+ alone	2.89 (1.25–6.71)	0.014^*^
MRDco+	6.10 (2.09–17.78)	0.001^*^
DFS		
Cytogenetics and molecular abnormalities		
Intermediate	1.00	
Unfavorable	2.49 (1.32–4.72)	0.005^*^
Disease status before allo-HSCT		
CR1	1.00	
CR2	1.48 (0.51–4.27)	0.471
NR	6.23 (2.06–18.81)	0.001^*^
MRD status after allo-HSCT		
MRD-	1.00	
WT1+ alone	1.79 (0.88–3.65)	0.110
MRDco+	4.75 (2.00–11.29)	< 0.001^*^
OS		
Cytogenetics and molecular abnormalities		
Intermediate	1.00	
Unfavorable	2.42 (1.24–4.73)	0.010^*^
MRD status after allo-HSCT		
MRD-	1.00	
WT1+ alone	1.59 (0.74–3.38)	0.232
MRDco+	5.62 (2.34–13.52)	< 0.001^*^

*CR* Complete remission, *NR* No remission, *ISD* HLA-identical sibling donor, *URD* HLA-unrelated donor, *Haplo-RD* HLA-haploidentical related donor, *UCB* Umbilical cord blood, *MRD-* MRD negativity, *WT1+ alone* WT1 positivity alone, *MRDco+* Combined MRD positivity, *DFS* Disease-free survival, *OS* Overall survival, *HR* Hazard ratio, *CI* Confidence interval

* means *P* < 0.05

In total, 111 patients received haplo-RD in CR1. Among them, patients with WT1 positivity after allo-HSCT had a significantly higher 5-year CIR (34.8% vs. 4.0%; *P* < 0.001), a lower 5-year DFS (61.0% vs. 85.6%; *P* = 0.005), and a lower 5-year OS (64.4% vs. 85.5%; *P* = 0.017) than those with WT1 negativity. Eighteen patients received HLA-identical sibling donor (ISD) transplantation or URD, six of them relapsed, and three relapsed patients (50%) were WT1 positive. The other 12 patients did not relapse, and seven of them (58%) were WT1 positive.

The CIR of WT1 positive patients was compared with that of WT1 negative patients at each monitoring time point (1, 2, 3, 4.5, 6, 9, and 12 months post-transplantation). Patients with WT1 positivity at + 1 month (5-year CIR: 30.8% vs. 20.1%; *P* = 0.277), + 3 months (5-year CIR: 16.7% vs. 21.2%; *P* = 0.834), + 4.5 months (5-year CIR: 14.6% vs.19.6%; *P* = 0.646), and + 12 months (5-year CIR: 16.7% vs.2.1%; *P* = 0.067) were not likely to relapse more than those with WT1 negativity. Nevertheless, patients with WT1 positivity at + 2 months、 + 6 months、and + 9 months relapsed more than those with WT1 negativity. Patients with WT1 positivity at + 2 months had a significantly higher 5-year CIR (40.0% vs. 20.2%; *P* = 0.019), a lower 5-year DFS (46.7% vs. 75.4%; *P* < 0.001), and a lower 5-year OS (41.5% vs. 78.8%; *P* < 0.001) than those with WT1 negativity. Patients with WT1 positivity at + 6 months had a significantly higher 5-year CIR (28.6% vs. 14.3%; *P* = 0.035) than those with WT1 negativity, but the DFS and OS showed no difference between these two groups. Patients with WT1 positivity at + 9 months had a significantly higher 5-year CIR (32.5% vs. 3.9%; *P* = 0.002), a lower 5-year DFS (67.5% vs. 92.5%; *P* = 0.014), and a lower 5-year OS (70.3% vs. 94.3%; *P* = 0.036) than those with WT1 negativity.

### Preemptive intervention after allo-HSCT

**Fig. 4:**
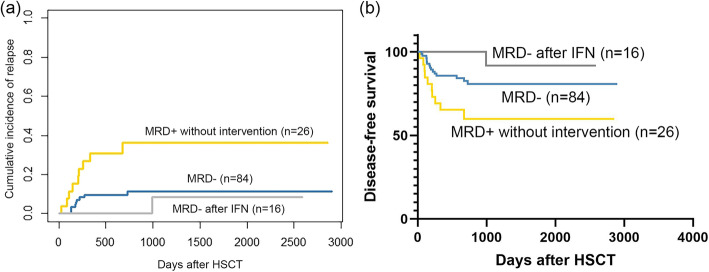
The outcomes of patients according to preemptive IFN-α treatment. **a** 5-year CIR: MRD- after IFN vs. MRD-: 8.3% vs. 11.3%, *P* = 0.513; MRD- after IFN vs. MRD+ without intervention: 8.3% vs. 36.2%, *P* = 0.024; MRD- vs. MRD+ without intervention: 11.3% vs. 36.2%, *P* = 0.004. **b** 5-year DFS: MRD- after IFN vs. MRD-: 91.7% vs. 80.8%, *P* = 0.208; MRD- after IFN vs. MRD+ without intervention: 91.7% vs. 59.9%, *P* = 0.014; MRD- vs. MRD+ without intervention: 80.8% vs. 59.9%, *P* = 0.019

Among the 67 patients who showed MRD positivity within 1 year after allo-HSCT (WT1+ alone: *n* = 54; MRDco+: *n* = 13), 25 received preemptive IFN-α treatment alone. Sixteen patients (64%) achieved MRD negativity after IFN-α treatment, with a median of 43 (range, 10 to 542) days from IFN-α treatment to MRD turning negative. One patient relapsed after achieving MRD negativity. The CIR (5-year CIR: 8.3% vs. 11.3%; *P* = 0.513) and DFS (5-year DFS: 91.7% vs. 80.8%; *P* = 0.208) were comparable between patients achieving MRD negativity after IFN-α treatment and those without MRD after allo-HSCT, which were both better than those of patients who had MRD but did not undergo any preemptive therapies (Fig. 4). Among the 9 patients who showed persistent MRD after IFN-α treatment, 3 relapsed, and the median time from IFN-α treatment to relapse was 70 days (range, 9 to 125).

Sixteen patients received preemptive Chemo-DLI (WT1+ alone: *n* = 10; MRDco+: *n* = 6), including 10 patients receiving IFN-α treatment prior to Chemo-DLI (persistent MRD after IFN-α treatment: *n* = 8; regained MRD positivity after achieving MRD negativity: *n* = 2). Four (25%) patients achieved MRD negativity after Chemo-DLI, with a median of 31.5 (range, 30 to 32) days from Chemo-DLI to MRD turning negative. Among the 4 patients achieving MRD negativity, 1 (25%) showed DFS after Chemo-DLI, 1 (25%) suffered from NRM, and 2 (50%) experienced relapse. Among the 12 patients who showed persistent MRD after Chemo-DLI, 7 (58.3%) experienced relapse.

### WT1 positivity over 1 year after Allo-HSCT

In total, 56 patients who achieved DFS without preemptive intervention had data of WT1 expression over 1 year after allo-HSCT. In these patients, the median WT1 expression levels at + 18, + 24, + 30, + 36, + 48, and + 60 months were 0.280% (0.020–1.100%), 0.245% (0.041–0.860%), 0.300% (0.042–1.500%), 0.260% (0.085–1.200%), 0.210% (0.055–0.370%), and 0.200% (0.015–1.300%), respectively (eFigure 3 in the Supplement). Four (4/56) patients were WT1 positive. Among them, two were transient WT1 positive, while the other two patients were persistent WT1 positive and had cGVHD.

In total, 31 patients relapsed after allo-HSCT. Among them, 25 patients relapsed within 1 year after allo-HSCT. Four patients received preemptive intervention within 1 year after allo-HSCT. Thus, only two relapsed patients had data of WT1 expression over 1 year after allo-HSCT and WT1 expression levels were both negative (< 0.8%) in these two patients.

Spearman correlation analysis showed that WT1 positivity was not associated with MFC positivity (Spearman’s correlation coefficient: -0.043, *P* = 0.744) over 1 year after allo-HSCT.

## Discussion

Our study observed that the relapse rate of pediatric AML patients who had WT1 expression ≥0.8% within 1 year after transplantation was significantly higher than that of patients with WT1 expression < 0.8%. Moreover, preemptive IFN-α treatment could help to clear MRD, decrease the risk of relapse, and improve DFS. To the best of our knowledge, our study shows the first relapse prediction result of WT1 sequential monitoring after allo-HSCT in a disease-specific population of children with AML, and these results provide an opportunity for exploring the up-to-date undefined predictive role of WT1 in these patients.

A previous study monitored WT1 expression at 2 time points after allo-HSCT (+42d and + 100d) and showed that WT1 could not predict relapse in pediatric AML [16]. Using only 2 time points to monitor WT1 may miss data of other important time points. Relapsed patients with normal WT1 expression at 2 time points measured may actually have elevated WT1 at other time points without detection. We performed WT1 monitoring at 1, 2, 3, 4.5, 6, 9, and 12 months post-transplantation, which was sequential monitoring and could lower the frequency of false negativity. Compared to the previous study [16], we monitored WT1 expression after 3 months post-transplantation additionally. Our data showed that WT1 monitoring at + 6 months、 + 9 months could also predict relapse, which suggested that monitoring WT1 expression after 3 months post-transplantation was also reasonable.

Based on ROC analysis, we defined WT1 positivity as WT1 ≥ 0.8%, while WT1 ≥ 0.6% was considered WT1 positivity in our previous studies enrolling adult patients [11, 12]. A previous study reported that children had a higher WT1 expression level than adults under physiological conditions [15]. Because WT1 was not a leukemia-specific molecular marker, a relatively higher cut-off value could spare pediatric AML patients who had slightly elevated WT1 levels but were actually in molecular remission from further interventions.

MRD could be monitored by MFC in AML, and MRD positivity was recommended to be defined as MFC > 0.1% [5]. Therefore, MRD detected by MFC might not be sensitive enough to predict relapse and direct preemptive intervention. In this study, we found that WT1 was significantly associated with MFC, and some patients showed WT1 positivity prior to MFC positivity with a median interval of 95 days. These results suggested that WT1 could reflect the residual leukemia cells in AML children and could be used as an MRD marker in these patients. Nevertheless, the association between WT1 positivity and MFC positivity was not so strong in the study. A large scale, multicenter study is needed to explore the relationship between WT1 and MFC in the future. In addition, we observed that patients with WT1+ alone had a trend of a lower CIR than those with MRDco+ (i.e., WT1+ and MFC+), which suggested that there might be a lower leukemia burden for patients with WT1+ alone. Because immunotherapy should preferably be started in patients with a relatively low leukemia burden [31], WT1-directed immunotherapy may help to clear MRD more promptly and effectively.

In order to remove the influence of population heterogeneity, we restricted prognostic analysis to patients who received haplo-RD in CR1. Results showed that patients with WT1 positivity had worse prognosis than those with WT1 negativity. This result was in consistent with that of the whole cohort. In patients who received ISD or URD, the ratio of WT1 positivity (50% vs. 58%) was not different between relapse and non-relapse groups. But it was hard to draw a conclusion about the prognostic role of WT1 expression in patients receiving ISD or URD because of the limited patients included in the study. Thus, in the future, a large scale and multicenter study is needed to address this issue.

Theoretically, the kinetics of MRD could predict prognosis. Since the influence of preemptive intervention should be removed, WT1 positive patients without preemptive intervention were used for analyses. Nine patients relapsed, and eight of them were WT1 positive once before relapse, while only one patient showed WT1 positive more than once before relapse. Thus, since there was only one relapsed patient with WT1 positivity more than once, we failed to explore the prognostic role of the kinetics of WT1 expression. A prospective, large scale and multicenter study is needed to explore the prognostic value of the kinetics of WT1 expression in the future.

WT1 was not a gold standard for starting preemptive intervention as relapse prediction of WT1 in pediatric AML after allo-HSCT was unclear. Doctors and patients started preemptive intervention depending on their own intention. However, many studies showed that WT1 could guide preemptive intervention in adult AML after allo-HSCT [11, 12, 23–30]. Therefore, we wanted to see whether pediatric AML patients could benefit from WT1-directed preemptive intervention. In patients with WT1 positivity determined by ROC analysis, we analyzed whether patients benefited from WT1-directed immunotherapy by comparing the outcomes of patients receiving preemptive intervention with those without it. We separated the whole patients into four groups (MRD negativity, MRD positivity without preemptive intervention, MRD positivity treated with IFN-α, MRD positivity treated with Chemo-DLI) to perform analysis, which may help to lower the impact of population heterogeneity on patients’ outcomes.

IFN-α treatment could clear MRD effectively [23, 25–28]. Our study found that 64% of pediatric AML patients achieved MRD negativity after preemptive IFN-α monotherapy. In addition, the CIR of patients who achieved WT1 negativity after IFN-α treatment was comparable to that of those without MRD after allo-HSCT, which was lower than that of WT1-positive patients without any preemptive interventions. These results were in accordance with those of our previous studies [23, 25–28]. It is suggested that IFN-α treatment could partially overcome the poor prognostic significance of increased WT1 expression after allo-HSCT, which further confirmed that WT1 could be used as an MRD marker in AML children. Several patients with persistent MRD after IFN-α treatment did not relapse. WT1 was not a leukemia-specific molecular marker. Thus, it was unavoidable that some patients may receive IFN-α treatment based on elevated WT1 which was not relative to leukemia in fact. However, IFN-α could reduce relapse in patients with WT1 positivity, which suggested that most patients could benefit from WT1-directed IFN-α treatment. In addition, IFN-α was safe and brought a very low risk of severe side effects. Few pediatric AML patients got MRD negativity nearly 2 weeks after IFN-α treatment. According to previous research [31], the earlier patients with positive MRD started immunotherapy, the better results they would get because of low leukemia burden at the time of preemptive intervention. Despite this, the median interval from IFN-α to MRD turning negativity was 43 days. Maybe there was false positivity of WT1, but this was the inevitable defect of any MRD detection method. Actually, our study showed that WT1 had relatively high sensitivity and specificity to predict relapse in pediatric AML patients after allo-HSCT.

It seemed that the efficacy of DLI was not as good as that observed in our previous studies [24, 29, 30]. This may be because most patients (62.5%) showed persistent MRD or regained MRD positivity after achieving negativity after prior IFN-α treatment. That is, Chemo-DLI was used as salvage therapy in these patients. Graft-versus-leukemia (GVL) is the critical mechanism of clearing MRD for both IFN-α and DLI, so an unsatisfactory response to IFN-α treatment suggests that residual leukemia cells may have immune escape and may not be sensitive to other immunotherapies (e.g., DLI) after allo-HSCT. Similarly, our previous study observed that the efficacy of salvage Chemo-DLI for IFN-α treatment was not sufficient [26]. Thus, how to further clear the MRD of these patients should be further studied. However, due to the small number of DLI cases, it would be premature to derive conclusions regarding the efficacy of Chemo-DLI in children with AML.

The prognostic role of WT1 expression and preemptive intervention directed by WT1 expression within 1 year after allo-HSCT had been discussed above. Although we also monitored WT1 expression over 1 year after allo-HSCT, the monitoring intervals were at least 6 months, which may not unveil the change of WT1 expression completely. Since patients included in the relapse group was not enough (*n* = 2), we failed to compare the WT1 expression levels between relapse and non-relapse groups. A prospective, large scale and multicenter study is needed to address this issue in the future.

Chimerism was not detected regularly at our center. The sensitivity of chimerism to predict relapse was lower than MRD, and mixed chimerism was not equal to relapse [32].

Our study had the following limitations. First, the study was a retrospective study. In addition, the number of patients included in the study was not large enough, particularly the number of patients receiving preemptive interventions. Thus, we could not further compare the efficacy between IFN-α treatment and Chemo-DLI in these patients. Future randomized, prospective trials may further compare the efficacy of these interventions. In addition, no patients received hypomethylating agents in the present study. These agents are worth further study, particularly for those who showed unsatisfactory responses to immunotherapies. Last, because the scale of our study was not much large, we were unable to perform test set analysis.

## Conclusions

In conclusion, WT1 sequential monitoring was able to predict relapse in pediatric AML patients after allo-HSCT. In addition, WT1-directed immunotherapy may have the potential to prevent relapse and improve survival in AML children after allo-HSCT.

## Supplementary Information

**Additional file 1: eMethods**. **eTable 1.** The sensitivity and specificity of different cut-off value for WT1**. eFigure 1.** ROC curve of WT1 expression level and the relapse rate (*n* = 96). **eFigure 2.** The outcomes of the total patients according to WT1 after allo-HSCT (*n* = 151). **eFigure 3.** WT1 expression at different points over 1 year after allo-HSCT in patients maintaining CR without interventions (*n* = 56).

## Data Availability

The datasets used and analyzed during the current study are available from the corresponding author on reasonable request.

## Abbreviations

AML: Acute myeloid leukemia
Ara-C: Cytarabine
BM: Bone marrow
CsA: Cyclosporine A
CR: Complete remission
CI: Confidence interval
CIR: Cumulative incidence of relapse
Chemo-DLI: Chemotherapy plus donor lymphocyte infusion
DFS: Disease-free survival
haplo-RD: HLA-haploidentical related donor
IFN-α: Interferon-α
LAIPs: Leukemia-associated aberrant immune phenotypes
MRD: Minimal residual disease
MFC: Multiparameter flow cytometry
MMF: Mycophenolate mofetil
MTX: Methotrexate
MP: Methylprednisolone
MRDco+: Combined MRD positivity
NRM: Non-relapse mortality
OS: Overall survival
PCR: Polymerase chain reaction
URD: HLA-unrelated donor
UCB: Umbilical cord blood
WT1: Wilms’ tumor gene 1
WT1+ alone: WT1 positivity alone
