# Supplementary material for: Wilms’ tumor gene 1 is an independent prognostic factor for pediatric acute myeloid leukemia following allogeneic hematopoietic stem cell transplantation
Source: BMC Cancer. 2021 Mar 19;21:292. doi: 10.1186/s12885-021-08022-0 (PMC7980537; doi:10.1186/s12885-021-08022-0)
Supplement: Supplementary file 1 — Additional file 1: eMethods. eTable 1. The sensitivity and specificity of different cut-off value for WT1. eFigure 1. ROC curve of WT1 expression level and the relapse rate (n = 96). eFigure 2. The outcomes of the total patients according to WT1 after allo-HSCT (n = 151). eFigure 3. WT1 expression at different points over 1 year after allo-HSCT in patients maintaining CR without interventions (n = 56). [file 12885_2021_8022_MOESM1_ESM.docx]

**Wilms’ tumor gene 1 is an independent prognostic factor for pediatric acute myeloid leukemia following allogeneic hematopoietic stem cell transplantation**

**Authors:** Dao-Xing Deng^1,#^, Juan-Juan Wen^1,2 #^, Yi-Fei Cheng^1^, Xiao-Hui Zhang^1^, Lan-Ping Xu^1^, Yu Wang^1^, Chen-Hua Yan^1^, Yu-Hong Chen^1^, Huan Chen^1^, Wei Han^1^, Feng-Rong Wang^1^, Jing-Zhi Wang^1^, Ya-Zhen Qin^1^, Kai-Yan Liu^1^, Xiao-Jun Huang^1,3,4^, Xiao-Su Zhao^1,4*^, and Xiao-Dong Mo^1*^

**Affiliations**: ^1^ Peking University People's Hospital, Peking University Institute of Hematology, National Clinical Research Center for Hematologic Disease, Research Unit of Key Technique for Diagnosis and Treatments of Hematologic Malignancies, Chinese Academy of Medical Sciences, 2019RU029, Beijing Key Laboratory of Hematopoietic Stem Cell Transplantation, Beijing, China;

^2^Department of Hematology, Peking University Shenzhen Hospital, Shenzhen, China.

^3^Peking-Tsinghua Center for Life Sciences, Beijing 100871, China.

^4^Collaborative Innovation Center of Hematology, Peking University.

**^*^Correspondence**: Prof. Xiao-Dong Mo; Peking University People’s Hospital, Peking University Institute of Hematology, No. 11 Xizhimen South Street, Xicheng District, Beijing 100044, China; Tel:8610-8832-6001; Fax 8610-8832-4577; E-mail: [mxd453@163.com](mailto:mxd453@163.com); Prof. Xiao-Su Zhao: Peking University People’s Hospital, Peking University Institute of Hematology, No. 11 Xizhimen South Street, Xicheng District, Beijing 100044, China; <Tel:8610-8832-4576>; Fax 8610-8832-4577; E-mail: [zhao.xiaosu@outlook.com](mailto:zhao.xiaosu@outlook.com).

^#^Dao-Xing Deng and Juan-Juan Wen contributed equally to this work.

**eMethods.**

**Transplantation regimens** (Page 3)

**MRD monitoring and definition** (Page 3)

**Preemptive IFN-α treatment protocol** (Page 3)

**Preemptive Chemo-DLI protocol** (Page 3-4)

**Treatment of GVHD after preemptive intervention** (Page 4)

**References** (Page 4)

**eTable 1. The sensitivity and specificity of different cut-off value for WT1**

(Page 5)

**eFigure 1. ROC curve of WT1 expression level and the relapse rate (n =96).** (Page 6)

**eFigure 2.** **The outcomes of the total patients according to WT1 after allo-HSCT (n =151).** (Page 7)

**eFigure 3. WT1 expression at different points over 1 year after allo-HSCT in patients maintaining CR without interventions (n =56).** (Page 8)

**eMethods.**

**Transplantation regimens**

The preconditioning regimens consisted of cytarabine (Ara-C), busulfan (BU, 3.2 mg·kg^−1^·day^−1^ administered intravenously on days −8 to −6) (day 0 being the first day of donor cell infusion), cyclophosphamide (CY, 1.8 g·m^−2^·day^−1^, days −5 to −4), and simustine (Me-CCUN, 250 mg^.^m^−2^, day −3). Ara-C was administered at 2 g·m^−2^·day^−1^ (day −9) to the human leukocyte antigen (HLA)-identical sibling donor (ISD) group, at 2 g·m^−2^·day^−1^ (days −10 to −9) to the HLA-unrelated donor (URD) group and umbilical cord blood (UCB) group, and at 4 g·m^−2^·day^−1^ (days −10 to −9) to the HLA-haploidentical related donor (haplo-RD) group. Rabbit anti-thymocyte globulin (ATG, 2.5 mg·kg^−1^·day^−1^, days −5 to −2; Sanofi, Paris, France) was administered to the URD, haplo-RD, and UCB groups. In addition, recipients received cyclosporine A (CsA), mycophenolate mofetil (MMF), and short-term methotrexate (MTX) as GVHD prophylaxis. UCB transplantation recipients received methylprednisolone (MP) instead of MTX. Particularly, the patients with mother donors or collateral-related donors enrolled in the trial NCT02412423 received two doses of 14.5 mg/kg cyclophosphamide on days 3 and 4 post-HSCT.

**MRD monitoring and definition**

MRD monitoring was based on leukemia-associated aberrant immune phenotypes (LAIPs) detected by multiparameter flow cytometry (MFC), and Wilms’ tumor gene 1 (WT1) expression was determined through TaqMan-based RQ-PCR technology. A panel of eight antibody combinations that recognized CD7, CD11b, CD13, CD14, CD16, CD19, CD33, CD34, CD38, CD41, CD45, CD56, CD61, CD64, CD71, CD117, CD123, and HLA-DR was used for AML-LAIP detection. Isotype control monoclonal antibodies were used, and 0.2-1 million events per tube were routinely collected for analysis. Positive MRD was considered when a cluster of more than 20 cells with LAIP and SSC characteristics, identified in all plots of interest and carrying at least two LAIP markers identified at diagnosis, was observed. For those without LAIP markers at diagnosis, MRD was identified as a cell population showing deviation from the normal patterns of antigen expression seen on specific cell lineages at specific stages of maturation compared with either normal or regenerating marrow. The expression of *WT1* was evaluated by TaqMan-based real-time quantitative reverse transcription polymerase chain reaction (RQ-PCR). We selected ABL as a control gene. The experiments were performed in duplicate. The transcript level was calculated as *WT1* transcript copies/ABL copies in percentage. Routine MRD monitoring was performed 1, 2, 3, 4.5, 6, 9, and 12 months post-transplantation and at 6-month intervals thereafter. MRD positivity was defined as MFC positivity or WT1 positivity. WT1 positivity alone (WT1+ alone) was defined as WT1 positivity without MFC positivity in BM samples. Combined MRD positivity (MRDco+) was defined as both MFC positivity and WT1 positivity in BM samples.

**Preemptive IFN-α treatment protocol**

Recombinant human IFN-α-2b injections (Anferon; Tianjin Hualida Biotechnology Co., Ltd., Tianjin, China) were administered subcutaneously for 6 cycles (twice weekly in every 4-week cycle) at dosages of 3 million units for patients older than 16 years and at 3 million units per square meter for those younger than 16 years (capped by 3 million units). Prolonged treatment with IFN-α was permitted at the request of patients. MRD status was monitored 1, 2, 3, 4.5, 6, 9, and 12 months after preemptive IFN-α treatment and at 6-month intervals thereafter. Treatment with IFN-α was discontinued in any patient with active GVHD (grade II or higher acute GVHD or chronic GVHD with moderate or higher severity), severe infection, grade ≥ 3 toxicity, salvage Chemo-DLI use, relapse, or non-relapse mortality (NRM).

The patients who showed an unsatisfactory response to IFN-α treatment could receive salvage Chemo-DLI if they agreed to receive Chemo-DLI, did not have active GVHD, active infection, or organ failure. Patients who had positive MRD again after achieving MRD-negative status or those with persistent and increasing levels of MRD after preemptive IFN-α treatment were eligible for salvage Chemo-DLI.

**Preemptive Chemo-DLI protocol**

Our previous studies found that G-CSF-mobilized peripheral blood progenitor cells exerted stronger graft-versus-leukemia (GVL) effect without increasing the incidence of aGVHD [33-34]. Therefore, G-CSF–mobilized peripheral blood stem cells were administered instead of the more common unstimulated donor blood lymphocytes. Patients also received anti-leukemic chemotherapy 48–72 hours before DLI. The chemotherapy regimens included HAA (homoharringtonine, aclarubicin, and Ara-C), AA (aclarubicin and Ara-C), and HA (homoharringtonine and Ara-C). G-CSF–mobilized peripheral leukocytes were administered instead of unstimulated donor blood lymphocytes. The target dose of mononuclear cells was 1.0 × 10^8^/kg.

Patients received immunosuppressive drugs such as CSA or MTX to prevent GVHD after DLI. Patients receiving DLI from an ISD received GVHD prophylaxis for 4–6 weeks, while those receiving DLI from a haplo-RD or URD received GVHD prophylaxis for 6–8 weeks at the discretion of the attending physicians (and usually depending on the patient’s GVHD status after Chemo-DLI). The starting dosage of CSA was 2.5 mg·kg^−1^·day^−1^, which was adjusted to maintain a plasma concentration >100 ng/mL. MTX was administered at 10 mg intravenously on days 1, 4, and 8 and weekly thereafter for 2–6 weeks. MRD status was monitored 1, 2, 3, 4.5, 6, 9, and 12 months after preemptive Chemo-DLI and at 6-month intervals thereafter.

**Treatment of GVHD after preemptive intervention**

aGVHD was treated with methylprednisolone (1–2 mg∙kg^-1^ per day) and by the resumption of full-dose CSA administration. Second- or third-line immunosuppressive therapies such as CD25 monoclonal antibody (Basiliximab; Novartis Pharma Stein AG, Basel, Switzerland), MMF, tacrolimus, or MTX were administered in cases of steroid-refractory acute GVHD. Moderate to severe cGVHD was treated with prednisone (1 mg∙kg^-1^ per day), and CSA was adjusted to maintain a trough blood concentration >150 ng/mL. Second- or third-line immunosuppressive therapies such as MMF, MTX, penicillamine, azathioprine, rituximab, or tacrolimus were administered in cases of steroid-refractory cGVHD.

**References**

[33] Huang XJ, Wang Y, Liu DH, Xu LP, Liu KY, Chen H *et al*. Administration of short-term immunosuppressive agents after DLI reduces the incidence of DLI-associated acute GVHD without influencing the GVL effect. *Bone Marrow Transplant.* 2009;44(5):309-16.

[34] Yan CH, Liu DH, Xu LP, Liu KY, Zhao T, Wang Y *et al*. Modified donor lymphocyte infusion-associated acute graft-versus-host disease after haploidentical T-cell-replete hematopoietic stem cell transplantation: incidence and risk factors. *Clin Transplant*. 2012;26(6):868-76.

**eTable 1. The sensitivity and specificity of different cut-off value for WT1**

| WT1 Value | Sensitivity | Specificity | Youden's index |
| --- | --- | --- | --- |
| 0.61% | 66.7% | 69.1% | 0.358 |
| 0.80% | 60.0% | 79.0% | 0.390 |
| 1.05% | 40.0% | 85.2% | 0.252 |
| 1.35% | 26.7% | 91.4% | 0.181 |
| 1.70% | 26.7% | 93.8% | 0.205 |

**
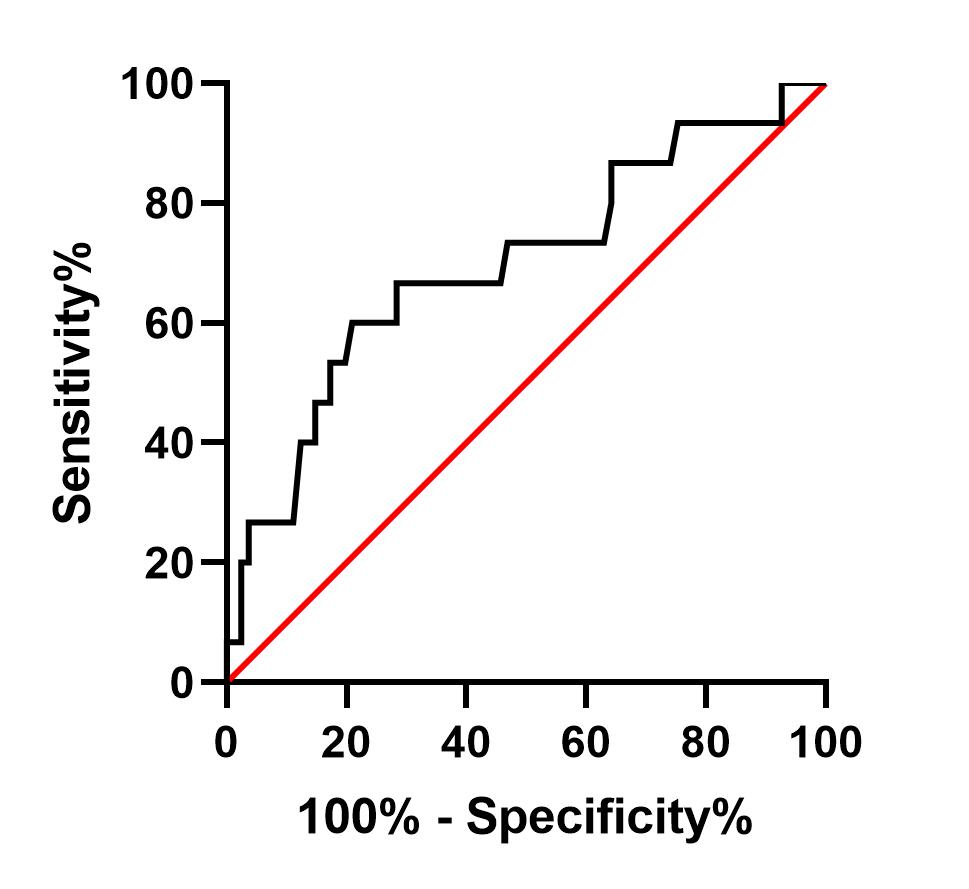
**

**eFigure 1.** **ROC curve of WT1 expression level and the relapse rate (n =96)**.


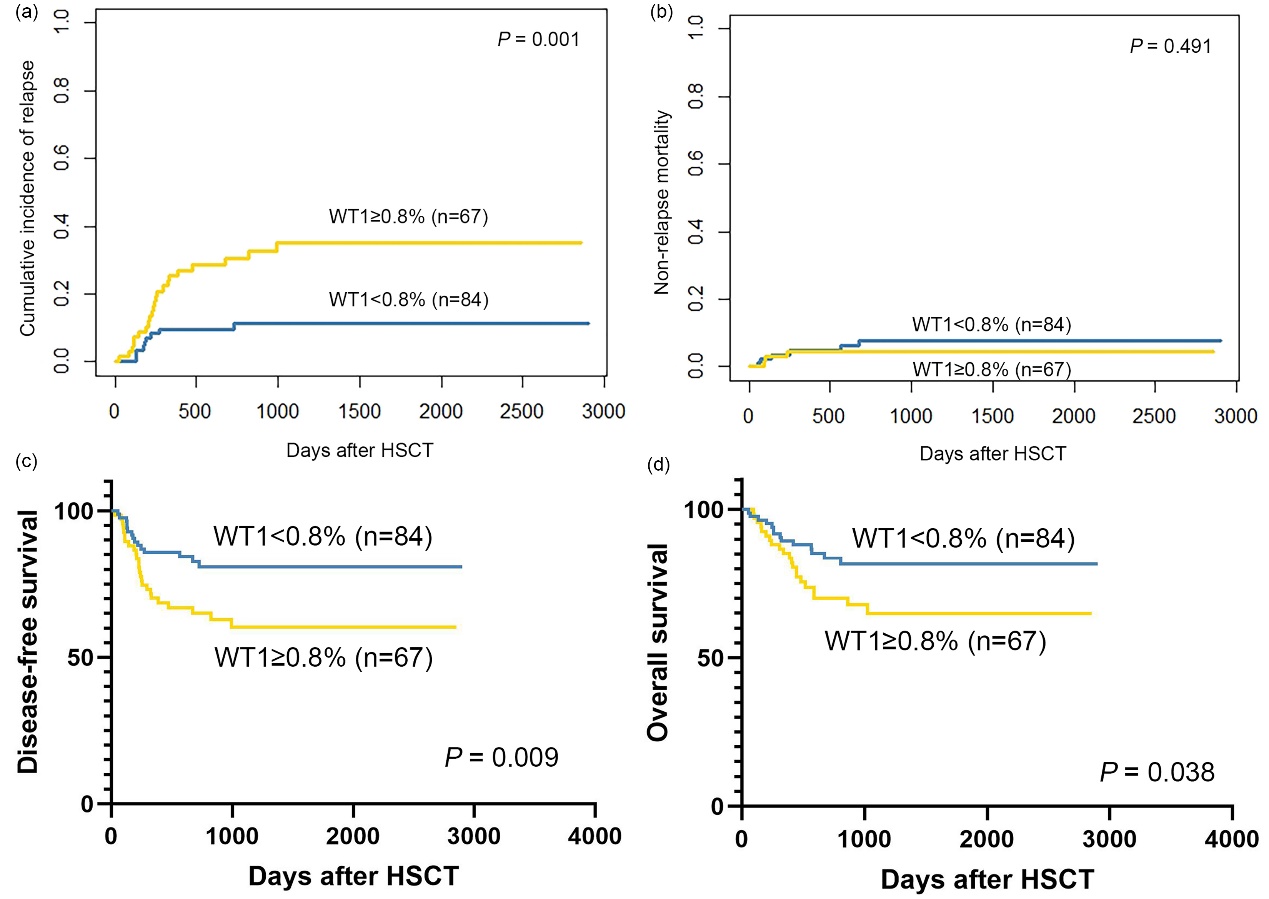


**eFigure 2.** **The outcomes of the total patients according to WT1 after allo-HSCT (n =151).** (a) relapse, (b) non-relapse mortality, (c) disease-free survival, and (d) overall survival.


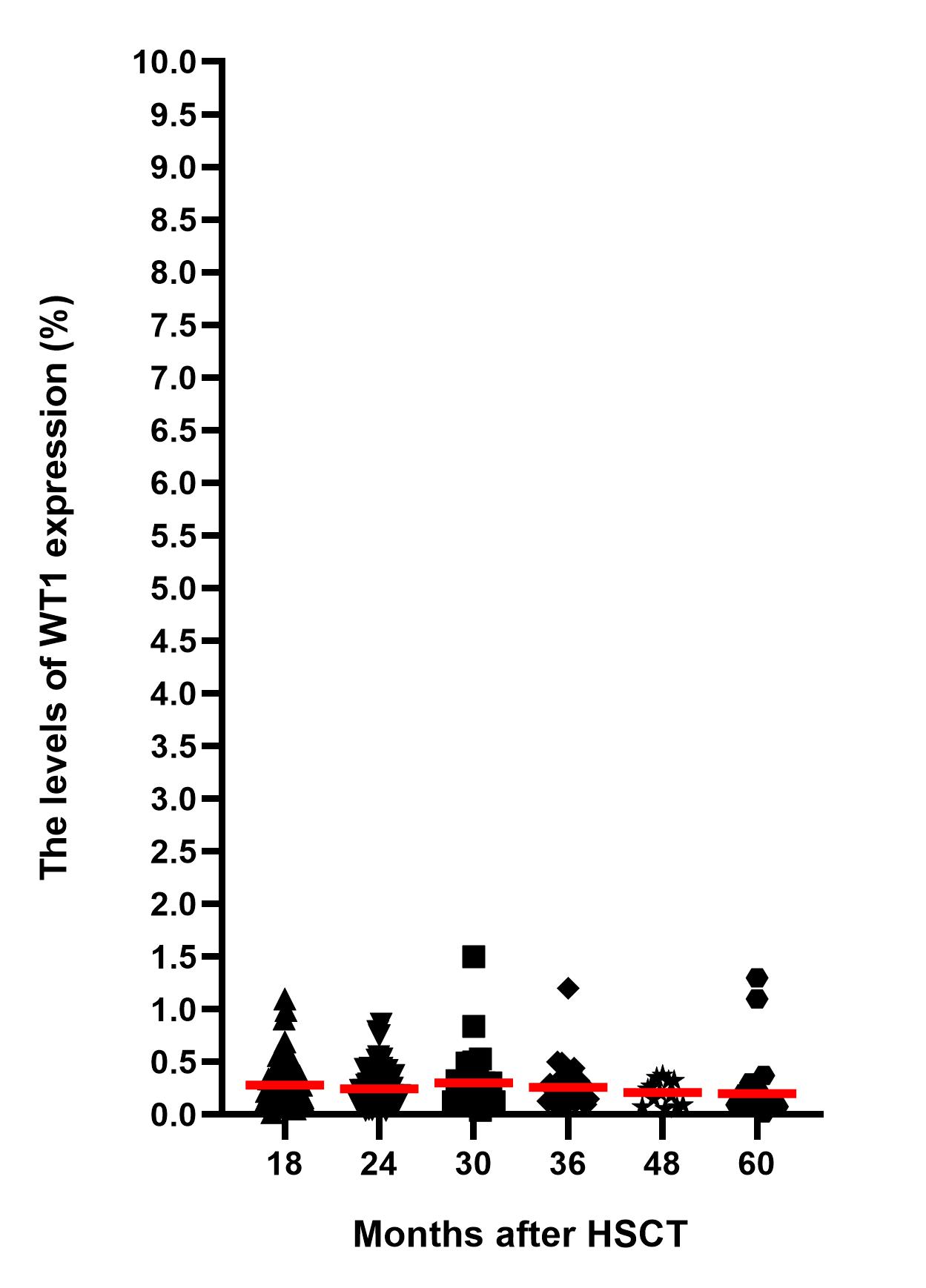


**eFigure 3.** **WT1 expression at different points over 1 year after allo-HSCT in patients maintaining CR without interventions (n =56).** Horizontal bars show the median values of WT1 at each time point.
